# Natural Killer Cell Immunomodulation: Targeting Activating, Inhibitory, and Co-stimulatory Receptor Signaling for Cancer Immunotherapy

**DOI:** 10.3389/fimmu.2015.00601

**Published:** 2015-12-02

**Authors:** Cariad Chester, Katherine Fritsch, Holbrook E. Kohrt

**Affiliations:** ^1^Division of Oncology, Department of Medicine, Stanford University, Stanford, CA, USA; ^2^Institute for Immunity, Transplantation and Infection, Stanford University School of Medicine, Stanford, CA, USA

**Keywords:** natural killer cells, immunotherapy, adoptive cell therapy, monoclonal antibody, cancer vaccines, checkpoint blockade

## Abstract

There is compelling clinical and experimental evidence to suggest that natural killer (NK) cells play a critical role in the recognition and eradication of tumors. Efforts at using NK cells as antitumor agents began over two decades ago, but recent advances in elucidating NK cell biology have accelerated the development of NK cell-targeting therapeutics. NK cell activation and the triggering of effector functions is governed by a complex set of activating and inhibitory receptors. In the early phases of cancer immune surveillance, NK cells directly identify and lyse cancer cells. Nascent transformed cells elicit NK cell activation and are eliminated. However, as tumors progress, cancerous cells develop immunosuppressive mechanisms that circumvent NK cell-mediated killing, allowing for tumor escape and proliferation. Therapeutic intervention aims to reverse tumor-induced NK cell suppression and sustain NK cells’ tumorlytic capacities. Here, we review tumor–NK cell interactions, discuss the mechanisms by which NK cells generate an antitumor immune response, and discuss NK cell-based therapeutic strategies targeting activating, inhibitory, and co-stimulatory receptors.

## Introduction

The recent FDA approvals of the programmed cell death protein 1 (PD-1)-targeted checkpoint inhibitors pembrolizumab and nivolumab mark the latest successes in the rapidly expanding field of cancer immunotherapies. Immunotherapy represents a paradigm shift in cancer treatment; instead of targeting tumor cells, the goal of immunotherapy is to augment and expand the immune system’s intrinsic antitumor response. To date, diverse immunotherapeutic modalities have been accepted as viable strategies for eliminating cancerous cells. Cytokines, cancer vaccines, adoptive cell transfers, and especially checkpoint inhibitors constitute valuable elements in the immunotherapeutic armamentarium. However, a class of important immune-modulators is conspicuously absent: agents that utilize the power of innate immune cells to eradicate tumors. An important class of innate immune cells that play a critical role in mediating the antitumor immune response is the natural killer (NK) cell.

First described in 1975, NK cells were initially identified as a distinct sub-population of lymphocytes by their capacity to spontaneously lyse tumor cells (1). NK cells are now accepted to play an important role in both the adaptive and innate immune responses that govern infection, autoimmunity, and tumor immunosurveillance (2, 3). Human NK cells are phenotypically characterized by the expression of CD56 and the absence of CD3 and can be further subdivided into a CD56^bright^ population and a CD56^dim^ population. The CD56^bright^ population produces immunoregulatory cytokines, including interferon-γ (IFN-γ), tumor necrosis factor-beta (TNF-B), tumor necrosis factor-α (TNF-α), granulocyte macrophage-colony stimulating factor (GMCSF), IL-10, and IL-13 (4). The CD56^dim^ subset is the terminally differentiated successor of the CD56^bright^ population and is primarily responsible for exerting cytolytic functions (5, 6). However, CD56^dim^ NK cells can produce cytokines, specifically IFN-γ, after cell triggering via NKp46 of NKp30 activating receptors or after stimulation with combinations of IL-2, IL-12, and IL-15 (7).

The defining functional feature of NK cells remains their intrinsic ability to conduct “natural killing” of cellular targets without prior sensitization. The antitumor effect provided by natural killing has been observed in tumors of hematopoietic and non-hematopoietic origins and reported in diverse *in vivo* models and clinical series (8). NK cell infiltration into tumor tissue is associated with better disease prognosis in colorectal cancer, clear cell renal cell carcinoma, and lung carcinomas (9–11). Additionally, a 11-year prospective cohort study of Japanese inhabitants linked low peripheral-blood NK cell cytotoxicity with increased cancer risk (12). The combination of compelling preclinical evidence and early clinical success has established NK cell immunotherapy as a promising therapeutic strategy in cancer. Here, we review the current understanding of the NK cell mechanisms underpinning antitumor immunity and discuss immunomodulatory targets for augmenting NK cell-mediated tumor clearance.

### Natural Killing

The initial hypothesis for the mechanism of NK cell-mediated killing postulated that the absence or altered expression of major histocompatibility complex (MHC) class I molecules would render target cells susceptible to NK cell attack (13). The “missing-self” hypothesis was the result of observations that NK cells can directly reject MHC class I-deficient tumors (14). Later *in vivo* experiments in murine and human systems confirmed that NK cytotoxicity was directly related to the absence of MHC class I expression on target cells (15, 16). However, the contemporary understanding of NK cell activation suggests that the transition of the NK cell from quiescence to activation is mediated by a network of activating and inhibitory receptors (17). While NK cells do express inhibitory receptors that detect the presence of MHC Class I molecules, it is the integration of multiple activating and inhibitory signals that determines if the NK cell becomes cytotoxic.

Natural killer cell cytotoxicity can be demonstrated in several related ways. The primary mechanism of cytotoxicity is based on granule exocytosis upon formation of an immunological synapse. NK cells contain preformed cytoplasmic granules that resemble secretory lysosomes and contain perforin and granzymes (18). Perforin is a membrane-disrupting protein that perforates the target cell membrane, while granzymes are a family of serine proteases that trigger cell apoptosis (19, 20). Upon activation, NK cells rapidly polarize the granules and reposition the microtubule organizing center toward the synapse with the target cell (21). The granule membrane then fuses with the plasma membrane, externalizes, and releases the cytotoxic granule contents, triggering target cell apoptosis (22).

NK cells can also contribute to target cell death indirectly by secreting pro-inflammatory cytokines. Two of the primary cytokines released by activated NK cells are IFN-γ and TNF-α. IFN-γ is a type II interferon that plays a critical role in promoting host resistance to microbial infection and protecting against tumor development (4). In the tumor microenvironment (TME), the IFN-γ released by NK cells stimulates CD4^+^ T cells to polarize toward a Th1 subset and accelerates the development of activated macrophages and cytotoxic, tumor-targeting CD8^+^ T cells (23). TNF-α is a multifunctional cytokine that can cause direct tumor necrosis by inflicting tumor-associated capillary injury, but also generates an adaptive immune response (24). TNF-α can enhance B cell proliferation and also promote monocyte and macrophage differentiation (25, 26). Together IFN-γ and TNF-α help to activate both innate and adaptive immune cells in the TME and generate a sustained antitumor immune response.

### Antibody-Dependent Cell-Mediated Cytotoxicity

Another granule-mediated mechanism of NK cell targeted killing is antibody-dependent cell-mediated cytotoxicity (ADCC). ADCC is thought to play an important role in mediating the antitumor effects of many of the monoclonal antibody (mAb) therapies used today as standard of care treatments for both solid tumors and hematologic malignancies (27). In ADCC, the Fc receptor expressed by NK cells (FcγRIII or CD16) binds to the Fc portion of the therapeutic antibody, which in turn is bound to tumor-associated antigen (TAA) on the tumor surface. The effectiveness of ADCC depends on the FcγRIII ligation on the NK cell. Patients with a FcγRIIIa polymorphism, resulting in high-affinity binding of FcγRIII to IgG1, demonstrate enhanced clinical benefit. This effect has been seen in patients treated with rituximab, trastuzumab, and cetuximab (28–30). ADCC was initially described as the release of cytotoxic perforin and granzyme by NK cells following ligation of FcγRIII by IgG target cells. However, ADCC is now recognized as a multi-tiered process that involves a network of coordinated immune cells and an adaptive immune response (31). For example, FcγR ligation on NK cells can induce the secretion of pro-inflammatory cytokines like IFN-γ, which can accelerate dendritic cell (DC) maturation (32). Mature DCs enhance antigen presentation and train tumor-specific lymphocytes, producing an immunological memory response (33).

### Death Receptor-Induced Apoptosis

Death receptor-induced apoptosis is a perforin-independent mechanism by which NK cells lyse target cells (34). This cytotoxic pathway relies on target cell expression of tumor necrosis factor (TNF) receptor superfamily members. The two main TNF receptors used in apoptotic induction are Fas (CD95) and TNF-related apoptosis-inducing ligand (TRAIL) (35). Fas is expressed on a wide variety of tissues, but Fas ligand (FasL) expression is restricted to activated NK cells and cytotoxic T lymphocytes (CTLs) (36). Fas cross-linking induces nuclear condensation, membrane blebbing, and caspase activation (37). The initial optimism surrounding the Fas–FasL pathway as a means of tumor control has decreased following the observations that Fas is downregulated in a variety of cancers during tumor progression (38).

TNF-related apoptosis-inducing ligand-mediated signaling is another death receptor-induced mechanism NK cells employ to kill target cells. TRAIL is constitutively expressed on some populations of NK cells and TRAIL-mediated signaling can induce spontaneous cytotoxicity against TRAIL-sensitive tumor cells (39, 40). Binding of TRAIL to its receptor, TRAILR1 or TRAILR2, results in receptor oligomerization on the cell membrane and triggering of a pro-apoptotic signal through activation of caspases (41). Preclinically, recombinant forms of TRAIL and agonistic anti-TRAIL receptor antibodies can have single-agent activity against TRAIL-sensitive tumor cells *in vitro* and *in vivo* (42). Recently, artificial nanoparticles coated with bioactive TRAIL demonstrated cytotoxicity against primary leukemic cells from a patient with acute lymphocytic leukemia (ALL) (43). However, despite preclinical successes, clinical trials of TRAIL-based therapies have demonstrated little efficacy and tumors rapidly develop resistance mechanisms to TRAIL (44). A better understanding of how tumors evade targeting and removal by NK cells is needed to overcome immunosuppression in the TME.

### NK–Tumor Interactions

**Figure 1:**
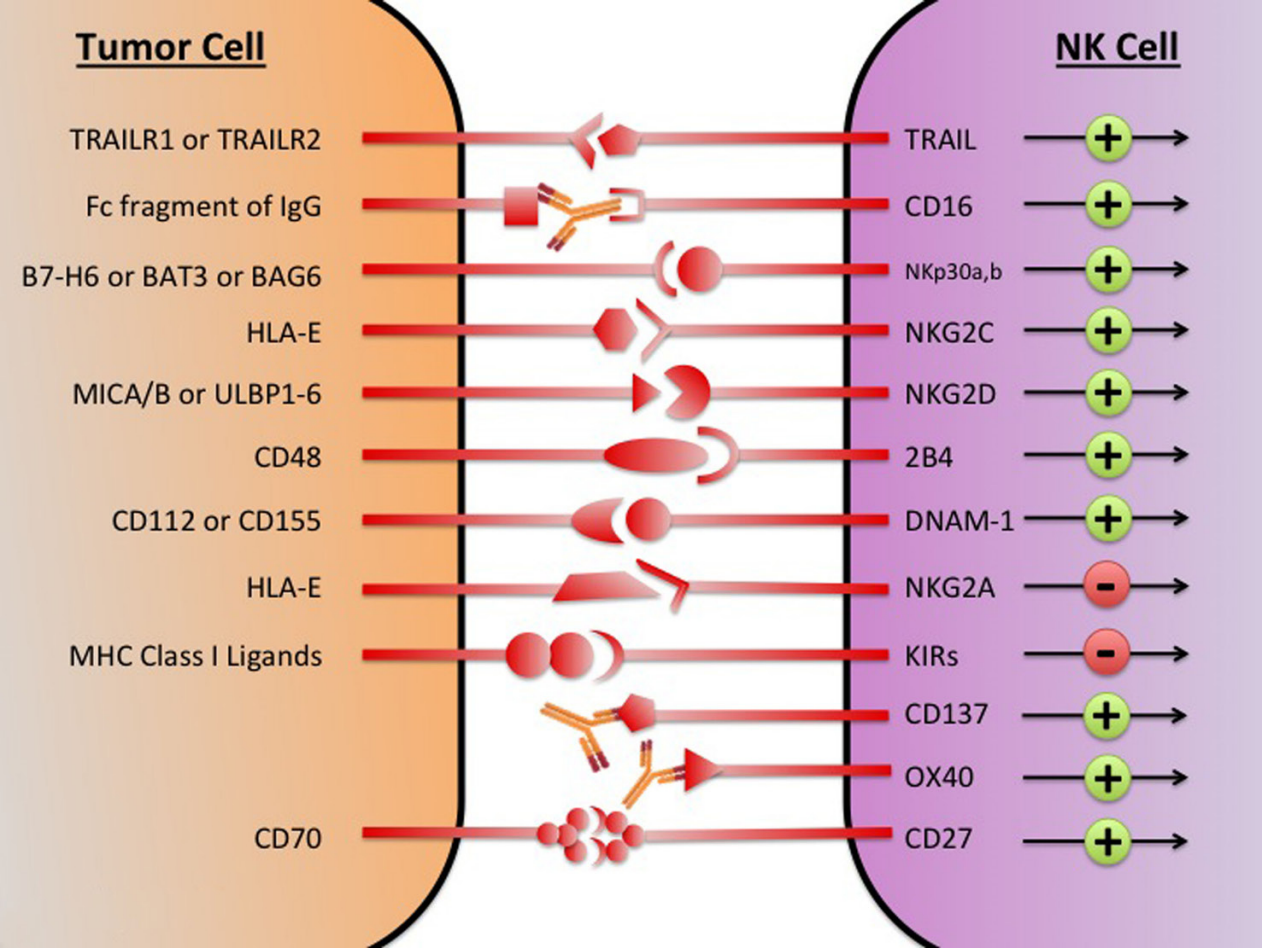
**The major NK cell receptors that are potential immunotherapeutic targets**. The transition of the NK cell from quiescence to activation is mediated by a network of activating and inhibitory receptors; it is the integration of the activating and inhibitory signals that determines if the NK cell becomes cytotoxic. Using immunotherapeutic agents to increase activation and decreases inhibitory signaling has the potential to generate NK cells with enhanced tumor lytic capacity. MICA/B, MHC class I chain-related proteins A and B; ULBP, UL16-binding protein; BAG, Bcl2-associated athanogene.

Despite the diverse repertoire of killing strategies utilized by NK cells, the tumor cell often avoids attack by direct and indirect mechanisms (45). Direct mechanisms consist of shedding soluble ligands for NK cell-activating receptors, upregulation of HLA molecules, and release of inhibitory cytokines. Indirect mechanisms consist of activation of inhibitory regulatory T cells (Tregs), DC killing, and phagocyte-derived inhibitory cytokines. These immunosuppressive mechanisms collectively create a TME where NK cell cytotoxic functions are inhibited. By stifling NK-mediated tumor eradication, the tumor escapes immunosurveillance and is able to grow and develop. Restoring and augmenting NK cell cytotoxic functions in the TME is an important step in overcoming immunosuppression and eliminating tumor. In an attempt to generate potent tumor-lysing NK cells, therapeutics are being developed that target NK cell activating, inhibitory, and co-stimulatory receptors (Figure 1).

## Activating Receptors

Activating receptors are a crucial element in regulating NK cell function. In the last decade, researchers have identified major signaling axes that control NK cell activation and suggested novel routes for therapeutic interventions (46). Some of the dominant activating receptors on NK cells are NKG2D, signaling lymphocytic activation molecule (SLAM) family molecule 2B4 (CD244), the DNAX accessory molecule (DNAM-1, CD226), and the NCRs: NKp30, NKp44, NKp46, and NKp80 (42). Recent work suggests that in NK cells, there is not a dominant receptor for activation, but instead receptors induce activation through combinatorial synergy (17). Only when multiple activating receptors are simultaneously engaged does the resulting signal surpass the requisite activation threshold and trigger cytokine secretion or direct cellular cytotoxicity. The requirement for activating receptor combinations helps prevent unrestrained activation of NK cells and provides flexibility in sensing and responding to environmental stimuli. What follows is a brief exploration of the dominant NK cell-activating receptors and summaries of attempts to target their tumorlytic capacity therapeutically.

### NCRs

All NK cells express NKp30 and NKp46, whereas NKp44 is only expressed on activated NK cells (47, 48). The acquisition of NCR during NK cell maturation correlates with the acquisition of cytolytic activity against tumor target cells (49). Inversely, downregulation of NKp30, NKp44, and NKp46 correlates with low NK cytolytic activity (50). NKp80 is expressed by virtually all fresh NK cells and mAb-mediated cross-linking of NKp80 resulted in induction of cytolytic activity and Ca2^+^ mobilization (51).

The NKp30 activating receptor has emerged as a promising therapeutic target in multiple cancer histologies. Downregulation is observed in patients with cervical cancer and high-grade squamous intraepithelial lesions (52). In lymphoma and leukemia models, ligation of NKp30 has been shown to activate human NK cells, trigger degranulation, and increase cytotoxicity (53). In patients with gastrointestinal sarcoma, the NKp30 isoform predicts the clinical outcome; patients with the immunostimulatory NKp30a and NKp30b isoforms have increased survival relative to patients with the immunosuppressive NKp30c isoform (54). Recently, the expression of distinct forms of NKp30 has been linked to 10-year progression free survival in patients with high-risk neuroblastoma (NB) (55). In NB patients with metastatic disease, the percentage of CD3^−^CD56^+^ NK cells in the peripheral blood and bone marrow was significantly elevated relative to patients with localized tumors. Additionally, NKp30 expression in the bone marrow of patients with metastatic NB was lower than expression in patients with localized NB (55). The ligand for NKp30, B7-H6, was highly expressed in neuroblasts, and the serum soluble form of B7-H6 correlated with tumor load and disease dissemination. The authors conclude that NK cell modulating immunotherapeutics offer a promising strategy for treating NB patients and that antibodies neutralizing sB7-H6 serum molecules and antibodies targeting NKp30 are worth pursuing in future clinical development.

### NKG2D

NKG2D, a homodimeric activating receptor and member of the C-type lectin superfamily, is expressed by all NK cells and subsets of T cells (56). NKG2D serves as a major recognition receptor for detection and elimination of infected and transformed cells (57). Ligands of the human NKG2D receptor are the MHC I-related molecules MICA/MICB, and the UL16-binding proteins (ULBP-1 to ULBP-6) (57). These ligands are rarely expressed in healthy tissues but induced by various cellular stresses, such as DNA damage, heat shock, or cellular transformation. Primary tumors frequently express NKG2D ligands: NK cell killing of both an urothelial tumor cell line and a bladder cancer cell appeared to be triggered by NK cell detection of the NKG2D ligands MICA/MICB (58, 59). However, tumors have also developed mechanisms for NK cell evasion despite NKG2D ligand expression. One such mechanism is the systemic release of NKG2D ligands by tumors in cancer patients (60, 61). The secreted NKG2D ligand was believed to cause downregulation of NK cell-expressed NKG2D, thus, depriving the NK cell of an activating signal and facilitating tumor escape. Recently, evidence has emerged that demonstrates an activating, antitumor role for soluble NKG2D ligands. The high-affinity MULT1 mouse NKG2D ligand can stimulate NK cells and enhance antitumor activity (62). The NKG2D pathway is integral to immune surveillance and an active area of immunotherapy research.

### 2B4 and DNAM-1

One of the best-characterized NK cell activation receptors is 2B4, a member of the SLAM receptor family. The first data to suggest a role for 2B4 in regulating NK cell activation demonstrated that ligation of 2B4 by 2B4-specific antibodies induced IFN-y production *in vitro* and triggered NK cell-mediated cytotoxicity (63). Following the identification of the natural ligand for 2B4, CD48, researchers reported that target cell expression of CD48 augmented NK cell-mediated cytotoxicity (64). Researchers also reported significantly greater cytotoxic effects if 2B4 ligation was accompanied by ligation of DNAM-1 (65). DNAM-1 is an Ig-like family glycoprotein expressed on most human NK cells, monocytes, and T lymphocytes (66). Early support for DNAM-1 controlling NK cell activation was provided by Lanier and colleagues using DX11, an anti-DNAM-1 mAb (67). Blockade via DX11 inhibited the cytotoxicity of NK cells against an array of different tumor cell lines. CD112 and CD155, two nectin family proteins regulated by cellular stress, were soon identified as the ligands for DNAM-1 (68). CD155 and CD112 are expressed in a wide range of both solid and hematologic tumors (69). In patients with NB, expression levels of CD155 and CD112 correlate with tumor cells susceptibility to NK cell-mediated lysis (70). However, tumors have developed mechanisms for downregulating NK cell DNAM-1 and effecting NK cell immunosuppression (71). In the design of future NK cell-based immunotherapies, mechanisms for preserving activation receptor surface expression need to be considered. Additionally, combinations of synergistic activating receptor pairs, like DNAM-1 and 2B4, need to be taken into account.

## Checkpoint Blockade in NK Cells

Immune checkpoint blockade strategies have proven a powerful approach to cancer immunotherapy. By blocking the receptors that transmit inhibitory signals to effector immune cells, checkpoint blockade aims to reverse immune suppression and generate robust antitumor immune responses. The successes of ipilimumab (anti-CTLA-4 mAb) and nivolumab and pembrolizumab (anti-PD-1 mAbs) demonstrate the potential of this therapeutic strategy. Ipilimumab (Yervoy, BMS) was approved in 2011 for the treatment of unresectable or metastatic melanoma, and blocks the CTLA-4-mediated signaling in T cells (72). CTLA-4 is an inhibitory receptor that upon ligation sends a negative regulatory signal to the T-cell receptor (TCR), limiting T cell activation (73). Nivolumab (Opdivo, BMS) and pembrolizumab (Keytruda, Merck) target programmed cell death protein-1 (PD-1). PD-1 is upregulated on T cells following activation and ligation of PD-1 transmits a negative regulatory signal (74). Histologically, diverse tumors upregulate the ligands of PD-1, PD-L1, and PD-L2 to take advantage of this immunosuppressive signaling pathway (75). Analogously to negative regulators of T cell activity, NK cells express surface receptors that can be targeted in checkpoint blockade strategies.

### Killer Cell Immunoglobulin-Like Receptor

Within the signaling pathways that govern NK lytic capacity, the killer cell immunoglobulin-like receptor (KIR) family is a dominant group of negative regulators. KIR receptors bind to the self-MHC class I ligands (HLA-A, -B, -C) and upon ligation transmit signals that abrogate the effects of activating receptors (76). The prevalence of MHC I on healthy cells provides an inhibitory signal that prevents NK cells from inducing autoimmune responses. However, in acute myeloid leukemia (AML) patients following haploidentical stem cell transplantation from KIR mismatched donors, the absence of KIR–HLA class I interactions resulted in potent NK cell-mediated antitumor efficacy and increased survival (77, 78). The antitumor effect can also be obtained without undergoing stem cell transplant; mAb therapy provides a viable route for blocking KIR–HLA interactions. Preventing HLA ligation to KIRs with an anti-KIR mAb has been shown to increase NK cell degranulation, IFN-γ secretion, and tumor cell lysis as well as increasing overall survival in murine cancer models (79).

The development of a candidate anti-KIR antibody had to overcome significant challenges. The KIR gene content varies substantially from individual to individual depending on the inherited KIR haplotype and the KIR family is composed of several structurally different proteins, necessitating an antibody that has cross-reactivity between different KIRs (80). Despite these challenges, the anti-KIR mAb lirilumab (Innate Pharma) has entered clinical trials. The initial phase I safety trial reported safety and potential efficacy in patients with AML (81). A second phase I trial confirmed the early reports of safety and durable KIR-blocking ability in patients with multiple myeloma (82). Recently, it has been reported that rituximab-mediated ADCC, a potent therapeutic mechanism of rituximab therapy, is reduced by KIR signaling (83). We have demonstrated that this KIR-mediated ADCC suppression can be overcome by combining rituximab with anti-KIR mAb therapy (84). Currently, multiple phase I and phase II clinical trials are ongoing, testing lirilumab (IPH2102/BMS-986015), as a monotherapy or in combination with other checkpoint inhibitors in patients with hematological and solid tumors (NCT01714739) and (NCT01750580).

### NKG2A

In addition to KIRs, the CD94/NKG2A heterodimer is another target for NK cell checkpoint blockade. The natural ligand of CD94/NKG2A is HLA-E, a non-classical HLA class I molecule that is expressed on the cell surface of most leukocytes and on transformed cells, including virus-infected cells and tumor cells (85, 86). Ligation of CD94/NKG2A by HLA-E transmits inhibitory signaling that suppresses the effector functions of NK cells, resulting in decreased cytotoxicity and cytokine secretion. HLA-E and CD94/NKG2A expression has been reported in multiple tumor histologies and is associated with poor prognosis. In colorectal cancer patients, tumor expression of HLA-E is associated with shorter disease-free survival time (87). In patients with head and neck squamous cell cancers (HNSCC), 78 to 86% of tumors express HLA-E (88). In patients with non-small cell lung cancer, intratumoral NK cells display higher expression levels of NKG2A mRNA relative to non-tumor NK cells (89). In breast cancer patients, expression of NKG2A by tumor infiltrating NK cells increases with cancer progression and correlates with impaired NK cell functions (90). Similarly to blocking KIR-mediated interactions, blockade of CD94/NKG2A-mediated signaling has the potential to restore and preserve NK cell cytotoxicity, leading to antitumor responses. A phase I/II trial testing an anti-NKG2A antibody (IPH2201, Innate Pharma) in HNSCC patients is ongoing (NCT02331875).

## Co-Stimulatory Signaling via mAbs

Activating co-stimulatory pathways to potentiate antitumor immune responses is a promising approach for augmenting NK-mediated tumor clearance. Members of the tumor necrosis factor receptor superfamily (TNFRsf) include several co-stimulatory proteins with key roles in the regulation of the activation, proliferation, and apoptosis of lymphocytes, including NK cells.

### CD137

First identified in 1989, CD137 (or 4-1BB) is a co-stimulatory receptor and member of the TNF receptor superfamily (91). CD137 is expressed on T cells and DCs and is upregulated on NK cells following FcγRIIIa ligation (92). In a variety of different tumor models, agonistic anti-CD137 mAbs have demonstrated the capacity to amplify antitumor immune responses and eliminate established tumors (93). Despite the broad expression of CD137 and its multiple contributions to immune dynamics, the therapeutic efficacy of anti-CD137 relies on functional NK cells. In preclinical models, the selective depletion of NK cells via the anti-AsialoGM1 or anti-NK1.1 antibodies completely abrogated the antitumor effect of anti-CD137 mAb therapy (94). Simultaneously, anti-CD137 agonistic antibodies increase NK cell proliferation, degranulation, and IFN-γ secretion, leading to enhanced ADCC of tumor cells (95). Because of the potential to enhance ADCC-mediated tumor clearance, anti-CD137 antibodies are being tested in combination treatment strategies with FDA-approved mAbs. We have previously demonstrated that antibodies targeting CD137 synergize with rituximab and trastuzumab to clear tumors in murine xenograft models of lymphoma and breast cancer (27, 96). Recently, we combined cetuximab and anti-CD137 antibody therapy to obtain complete tumor resolution and prolonged survival in xenograft models of EGFR-expressing cancer cells (97). In all three disease models and combination treatment regimens, expression of CD137 on NK cells increases significantly when NK cells encounter mAbs bound to tumor cells. We believe the synergy between anti-CD137 treatment and established mAbs demonstrates a promising therapeutic strategy and warrants future investigation.

Anti-CD137 mAb therapy has also entered clinical testing. The anti-CD137 antibody, urelumab, is currently in clinical trials with rituximab for patients with non-Hodgkin’s lymphoma (NCT01775631) and with cetuximab in patients with colorectal cancer or head and neck cancer (NCT02110082). In addition to urelumab, clinical trials of Pfizer’s anti-CD137 mAb, PF–05082566, are also ongoing (NCT01307267). A recent presentation of the preliminary findings reports that 27 patients with mixed tumor types have been treated with PF-05082566; disease stabilization was the best overall response, observed in 22% (6/27) patients (98).

### OX40

OX40, also known as CD134 or TNFRSF4, is a co-stimulatory molecule expressed primarily by activated T cells, but also expressed on natural killer T (NKT) cells and NKs (99). In NK cells, OX40 ligation appears to induce an activating signal and IFN-γ production (100). Engagement of the OX40 receptor *in vivo* in tumor-bearing mice enhanced antitumor immunity, resulting in increased survival in four separate murine tumor models of diverse histology and immunogenicity (101). The initial phase I/II trial of an anti-OX40 mAb demonstrated tolerability and regression of at least one metastatic lesion in 12 out of 30 study patients (102). Immunologically, treatment with agonistic anti-OX40 increased the proliferation of NK cells as well as CD4+ T cells (103). Additional trials of anti-OX40 are ongoing, including combination therapies with rituximab in patients with CLL and NHL (NCT01775631), with stereotactic body radiation in patients with metastatic breast cancer (NCT01862900), and with tremelimumab, an anti-CTLA-4 antibody, in patients with solid tumors (NCT02205333).

### CD27

In addition to its co-stimulatory role on T cells, the expression of CD27, or TNFRSF7, differentiates the NK cell compartment into two functionally distinct subsets. Circulating CD27^+^ NK have lower levels of perforin and granzyme B and demonstrate lower levels of cytotoxicity relative to CD27^-^ NK cells (104). The absence of CD27 expression in combination with the expression of CD11b is an indicator of cytolytic effector cells within human NK cell subsets. The natural ligand for CD27, CD70, induces downregulation of CD27 in a process controlled by the common γ-chain cytokine IL-15 (105). Signaling via CD27–CD70 interactions have been shown to accelerate NK-mediated tumor clearance while simultaneously stimulating cytokine secretion by NK cells that elicits an adaptive immune response (106).

The potential for CD27 ligation to generate an antitumor response has been confirmed in preclinical models. In a xenograft models of lymphoma, administration of the humanized anti-CD27 antibody, 1F5, significantly prolonged survival (107). The fully human 1F5 cannot bind to mouse CD27, therefore, any observed antitumor activity is attributed to effector mechanisms such as direct inhibition/apoptosis via CD27 signaling in tumors or ADCC. In syngeneic colorectal and lymphoma models with little to no expression of CD27, treatment with the 1F5 mAb also elicited antitumor activity and increased survival (108). By testing an aglycosylated version of the 1F5 mAb, the researchers demonstrated that FcR engagement was required for the antitumor effects of 1F5 therapy. An anti-CD27 mAb (Varlilumab or CDX-1127, Celldex Therapeutics) is currently being tested in a phase I trial in patients with solid tumors and hematologic cancers (NCT01460134). Preliminary findings report that of the 19 treated patients, 3 had stable disease and 1 had a complete readmission (109).

## Conclusion

In the future, immunotherapeutic agents that directly enhance NK cell-mediated tumor eradication will play a leading role in cancer treatment strategies. NK cells have novel mechanisms of participating in immune defense, making them uniquely appealing for cancer immunotherapy. Enhancing NK cell tumorlytic capacity is also a compelling combinatorial treatment strategy and would complement current standard of care treatments based on mAb therapy. The potential for NK-targeted agents to augment the antitumor effects of T cell checkpoint blockade is actively under consideration. As NK cell-based therapies move into the clinic, identifying prognostic biomarkers in the treatment populations will be crucial to the rational design of clinical studies. Concurrently, a greater effort must be made to profile the effects of novel immunotherapeutic agents, like checkpoint inhibitors, on NK cell function. The NK cell is now accepted as an integral part of the immunologic antitumor response. A number of promising NK-targeting therapeutics are in early-phase trials, and the results are eagerly awaited.

## Conflict of Interest Statement

The authors declare that the research was conducted in the absence of any commercial or financial relationships that could be construed as a potential conflict of interest.
